# Enhancing Hydrogen Evolution Catalysis through Potential-Induced Structural Phase Transition in Transition-Metal Dichalcogenide Thin Sheets

**DOI:** 10.1021/acs.jpclett.3c03305

**Published:** 2024-02-22

**Authors:** I-Wen Peter Chen, Yi-Lun Tseng, Jeremiah Hao Ran Huang, Kuan-Lun Chen, Tsai Yun Liu, Jui-Chin Lee

**Affiliations:** † Department of Chemistry, National Cheng Kung University, Tainan 701, Taiwan; ‡ Core Facility Center, National Cheng Kung University, Tainan 701, Taiwan; § Department of Applied Sciences, National Taitung University, Taitung 950, Taiwan

## Abstract

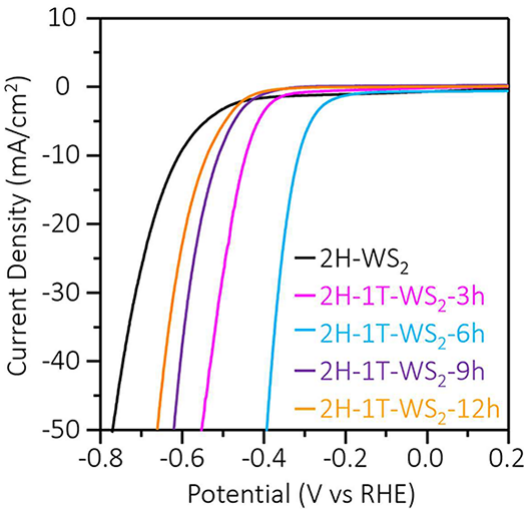

Enhancing electrocatalytic performance relies on effective phase control, which influences key catalytic properties, such as chemical stability and electrical conductivity. Traditional methods for manipulating the phase of transition-metal dichalcogenides (TMDs), including high-temperature synthesis, Li intercalation, and doping, involve harsh conditions and energy-intensive processes. This study introduces an innovative approach to crafting heterophase structures (2H-1T-WS_2_) in TMDs, using WS_2_ as a model compound, encompassing both semiconducting (2H) and metallic (1T) types through a straightforward potential activation method. Insights from in situ electrochemical Raman spectroscopy, HR-TEM, and XPS measurements reveal distinctive partial phase-transition behavior. This behavior enables the partially exposed basal plane of 2H-1T-WS_2_ to demonstrate superior activity in the hydrogen evolution reaction (HER), attributed to enhanced electrical conductivity and the exposure of highly active sites. The potential-induced phase transition presents promising avenues for the development of catalysts with heterophase structures.

The crystal structure of transition-metal dichalcogenides (TMDs) shows numerous crystal phases with distinct electrical, optical, and catalytic properties.^1,2^ Tuning structural changes between these different structural phases may provide a means of heterophase properties, with implications for potential applications.^3,4^ Structural phase changes in TMDs have so far been induced by pressure, electrostatic, chemical, and thermal routes.^5−9^ TMDs can exist as different polymorphs including 2H (semiconducting) and 1T (metallic) phases, relying on the coordination properties between the transition metal and chalcogen atoms.^10,11^ Importantly, the 1T-phase TMDs show promising behavior for catalytic hydrogen generation and energy storage compared to the 2H-phase TMDs because of the significant charge transfer resistance (*R*_ct_) reduction in the metallic phase.^12,13^ Therefore, to date, much research has focused on the production of 1T-TMD layers based on the 2H-TMD layers conversion reaction, which was prepared by light irradiation, electron beam, and metal intercalation/doping.^7,14,15^ Unfortunately, to understand the phase conversion process through the metal intercalation/doping method usually requires severe experimental conditions such as high temperature, high pressure, highly reactive reagents, and a precise chemical vapor deposition (CVD) technique under critical conditions to prepare the 1T phase of TMDs.^13,16^ However, the 1T phase of TMDs is not a thermodynamically stable form under ambient conditions. Recently, Liu et al. synthesized 2H-1T TMDs, and their heterostructure demonstrated thermodynamic stability and enhanced electrocatalytic performance.^17^ In-plane heterophases of MoS_2_ prepared by Bi et al.,^7^ red phosphorus vapor was effectively inserted into the interlayer of 2H-MoS_2_ bulk to generate partial phase transition from 2H to 1T phases of MoS_2_ bulk via high-temperature (1000 °C) vapor-doping process. The heterophases of MoS_2_ bulk exhibited a stable in-plane structure with excellent electrocatalytic performance, indicating nearly stable thermodynamic behavior. In the family of TMDs, MoS_2_ has been the most widely explored electrocatalyst. However, according to theoretical calculation results, WS_2_ is predicted to be a better active catalyst than MoS_2_.^18^ Moreover, WS_2_ has been less explored as an electrocatalyst, even though it potentially exhibits superior hydrogen evolution reaction (HER) performance compared to MoS_2_.^16,19^

In this study, we experimentally demonstrate that the electrochemical potential can effectively induce the 2H phase into the mixed 2H-1T phase of exfoliated WS_2_ thin sheets, resulting in the formation of in-plane structures with 1T and 2H WS_2_ domains. The phase transformation from 2H-WS_2_ to heterophases of 2H-1T-WS_2_ was monitored by in situ electrochemical Raman spectroscopy. Such novel in-plane 2H-1T-WS_2_ heterophase structures exhibit a highly stable electrochemical behavior and a superior charge transport property. Coupled with many exposed active sites, the 2H-1T-WS_2_ heterophase structure exhibits a low Tafel slope of 36 mV/decade, a high electrochemical active surface area (ECSA), and superior stability performance (2000 cycles) for HER.

Figure 1 shows a schematic of the chlorophyll-assisted exfoliated 2H-WS_2_ thin sheets via electrochemical potential stimulation to generate 2H-1T-WS_2_ heterophase structure.

**Figure 1:**
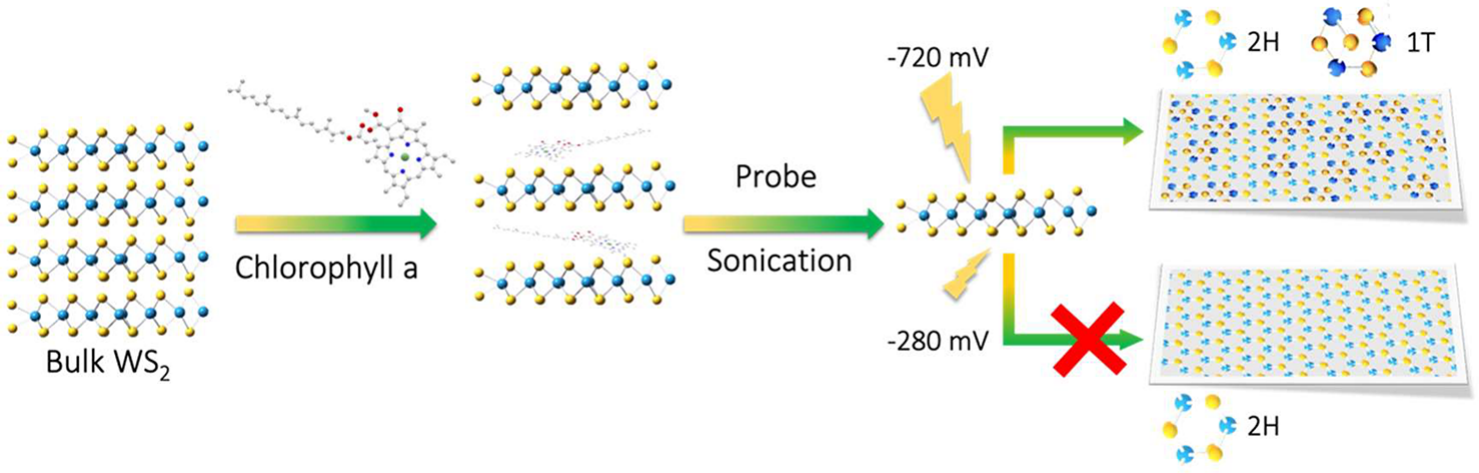
Schematic illustration of activation of TMDs (WS_2_ thin sheets) via an electrochemical potential application.

As illustrated in Figures 2a and 2b, the chlorophyll-assisted exfoliated thin sheets of high-quality WS_2_ can be successfully prepared via the liquid phase exfoliation route. Figure S1 shows that the thickness of the exfoliated WS_2_ thin sheets is around 1.3 nm. Figures 2c and 2d show the Raman and UV–vis spectra of the as-prepared WS_2_ thin sheets, which are in the 2H phase. The results aligned well with the 2H phase characteristic of the WS_2_ in the literature.^20−22^ The HER with 2H-WS_2_ thin sheets as the electrocatalyst on GCE was measured using a standard three-electrode electrochemical system in 2 M H_2_SO_4_ electrolyte. The polarization curve (without *iR* compensation) showing the current density versus potential (V vs RHE) for 2H-WS_2_ thin sheets is shown in Figure 2e. To investigate catalyst stability under electrocatalytic operation, we have measured the HER characteristics of 2H-WS_2_ thin sheets by monitoring the current density during continuous operation at −0.28 V (vs RHE) for 3 h, as shown in Figure 2f. Under this applied potential condition, the electrocatalytic performance of 2H-WS_2_ is stable. However, to achieve practical electrocatalytic application, the hydrogen generation of the as-prepared catalyst should be tested under a high-current-density condition.

**Figure 2:**
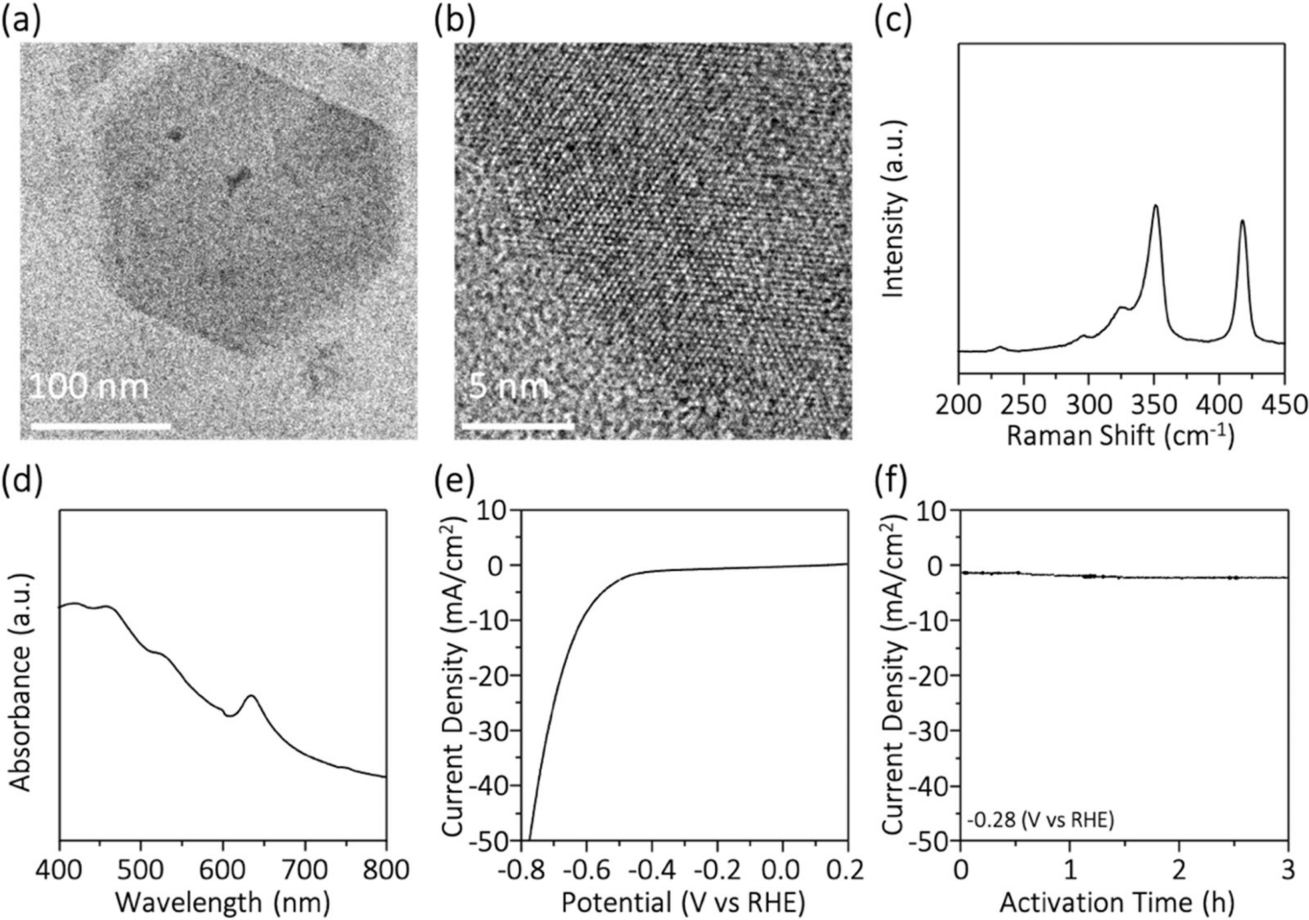
Structure and HER electrocatalytic properties of the chlorophyll-assisted exfoliated 2H-WS_2_ thin sheets: (a) low- and (b) high-resolution TEM images, (c) Raman spectrum, (d) UV–vis spectrum, (e) polarization curve, and (f) stability testing.

To study the catalyst stability at high current density conditions, a chronoamperometric (*i*–*t*) curve was recorded for the as-prepared 2H-WS_2_ thin sheets at a constant potential of −720 mV in a 2 M H_2_SO_4_ solution over 8.5 h. Surprisingly, Figure 3a shows that the HER current density of the 2H-WS_2_ catalyst continuously increases with time, indicating continuous catalyst activation at high electrochemical potential. Therefore, we monitored the polarization curve for various activation times, as shown in Figure 3b. After continuous operation at −720 mV for 6 h, the HER performance of the 2H-1T-WS_2_-6h sample shows significant enhancement. The overpotential difference between 2H-WS_2_ and 2H-1T-WS_2_-6h is 380 mV at −50 mA/cm^2^. Figure 3c shows that the Tafel slope of the 2H-1T-WS_2_-6h sample is 36 mV/decade, which is comparable to the state-of-the-art, Pt. Figure 3d shows that the overpotential of the 2H-1T-WS_2_-6h catalyst can be down to 0.31 V at −10 mA/cm^2^.

**Figure 3:**
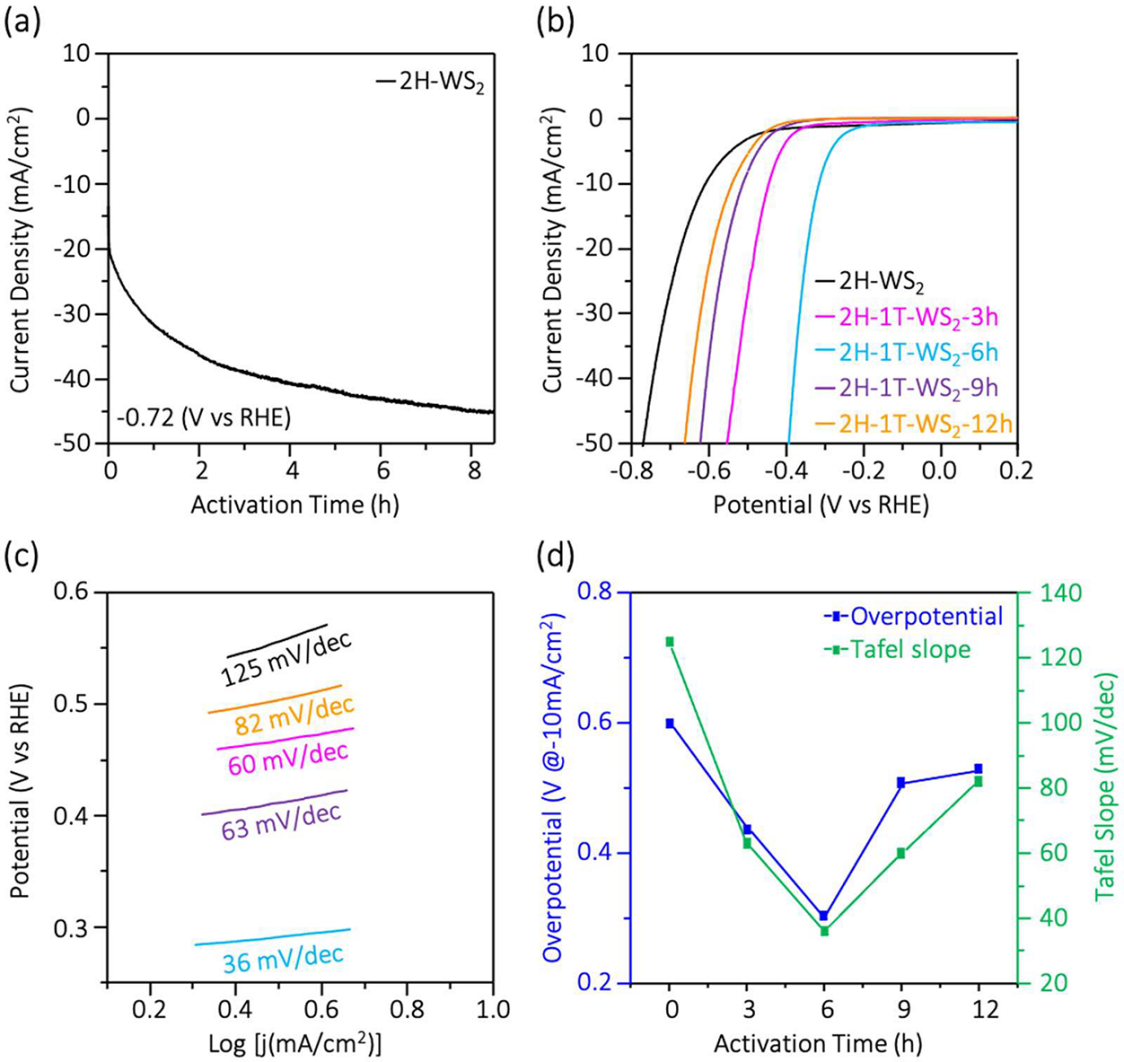
Activation-dependent HER measurements of WS_2_ thin sheets during continuous operation at −720 mV: (a) *i*–*t* curve, (b) polarization curves obtained with various activation times, (c) Tafel plots converted from the polarization curves in (b), and (d) summary of the value of the overpotentials and Tafel plots after different activation times.

To unveil the morphology and microstructure of activated WS_2_, scanning electron microscopy (SEM) and transmission electron microscopy (TEM) were utilized. The SEM images of the exfoliated WS_2_ thin sheets (Figure S2a) and the 2H-1T-WS_2_-6h sample (Figure S2b) revealed that the morphology of the samples before and after electrochemical activation treatment were similar, indicating that the structure of the exfoliated WS_2_ remained the same after electrochemical activation. However, to unveil the fine structure of samples before and after electrochemical activation treatment of WS_2_, high-resolution electron microscopy measurements were conducted. High-resolution TEM image (Figure 4a) revealed that the structure of the 2H-1T-WS_2_-6h sample was uniform after electrochemical activation. The FFT (Figure 4b) of the TEM image showed the presence of both 2H and 1T phase lattice regions on the WS_2_ surface. Figure 4c shows that the interlayer distance of these thin sheets was 0.633 nm, consistent with the 1T phase of WS_2_, indicating the formation of heterophase domains.^13^Table S1 shows the composition of the 2H-1T-WS_2_-6h sample via TEM-EDS measurement. The atomic percentages of W and S were 3.7% and 7.0%, respectively. The ratio of W/S was ∼1.9, which means only limited oxidation occurred on the surface of WS_2_ during the electrochemical activation process. To further identify the crystalline phase and chemical compositions of 2H-1T WS_2_ heterophase structures, in situ electrochemical Raman and UV–vis spectroscopy analyses were conducted. The Raman spectrum of the 2H-WS_2_ thin sheet has two characteristic modes of vibration, namely, E_2g_^1^ (in-plane vibrations of the W–S bond with the W atoms and the S atoms vibrating in opposite directions) and A_1g_ modes (out-of-plane vibrations of the S atoms), which are located at 351 and 417 cm^–1^, respectively. As shown in Figure 4d, the intensities of the two characteristic modes of vibration continuously decrease with activation time, indicating phase transformation occurrences.^23−26^Figure S3 shows the magnified Raman spectrum of the 2H-1T-WS_2_-6h sample. Three additional peaks appeared at 177.4 cm^–1^ (J_1_), 203.3 cm^–1^ (J_2_), and 390.0 cm^–1^ (J_3_) which existed in the 2H-1T-WS_2_-6h sample but not in the exfoliated 2H-WS_2_ thin sheets.^23,27^ Moreover, Figure 4e shows that the absorption peaks of the 2H phase of exfoliated WS_2_ thin sheets (black curve) appeared at 526 and 634 nm. However, the absorption peaks of the 2H-1T-WS_2_-6h sample (blue curve) completely disappeared, indicating the in-plane structure of WS_2_ formation of a 1T phase.

**Figure 4:**
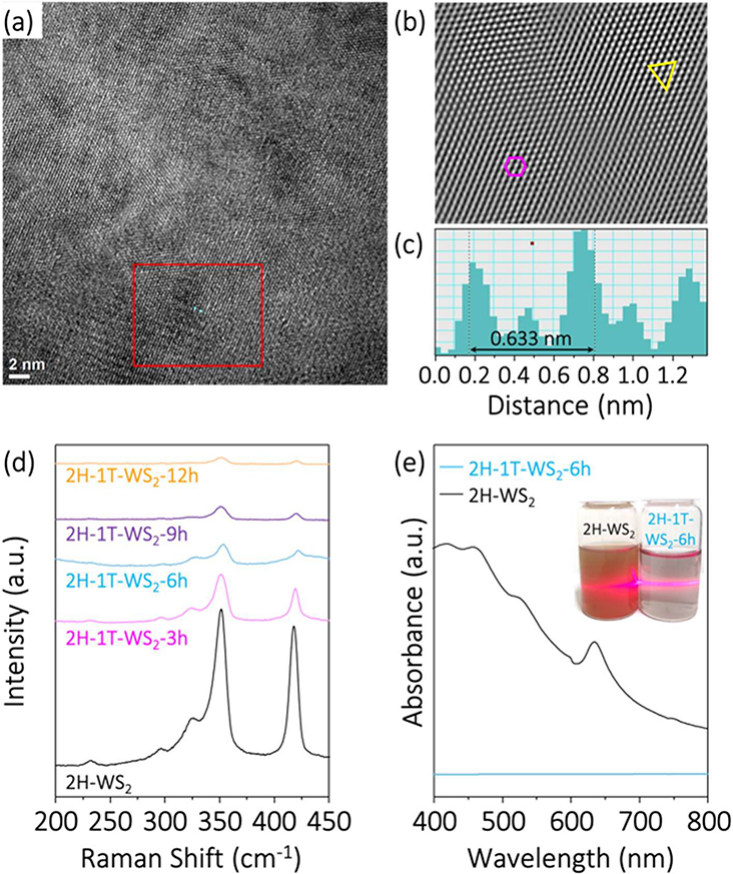
(a) TEM images of in-plane 2H-1T-WS_2_ heterophase structures. (b) FFT image of (a) red region and arrangement of 2H phase (pink hexagonal) and 1T phase (yellow trigonal). (c) The line profile shows the layer spacing in (a). (d) Raman spectra of the 2H-1T-WS_2_-3h, 2H-1T-WS_2_-6h, 2H-1T-WS_2_-9h, and 2H-1T-WS_2_-12h samples. (e) UV–vis spectra of the 2H-WS_2_ (black line) and 2H-1T-WS_2_-6h (blue line).

To characterize the composition of the electrochemically activated WS_2_ at the macroscale, we performed X-ray photoelectron spectroscopy. Figure 5 shows that the exfoliated 2H-WS_2_ thin sheet shows primarily the 2H phase with limited oxide formation. After applying potential to the exfoliated 2H-WS_2_ thin sheets for several hours, two new peaks appeared at 34.3 and 32.3 eV, which correspond to W^4+^ 4f_5/2_ and W^4+^ 4f_7/2_ of the 1T phase, respectively, as shown in Figure 5b–e. The 1T phase of the W 4f binding energy is lower than the 2H phase, which is similar to previous XPS results on 1T-MX_2_ materials (M: W or Mo; X: S).^16,23,28^Figure 5f summarizes that the integrated intensity of the 1T phase is 62.4% for 2H-1T-WS_2_-6h. When the activation time is increased to 9 and 12 h, the 1T phase of the WS_2_ thin sheets can be maintained around 50%. Nonetheless, the intensity of W^6+^ (WO_3_) increased significantly, and this could be one of the factors contributing to the reduction in the catalyst’s HER performance.

**Figure 5:**
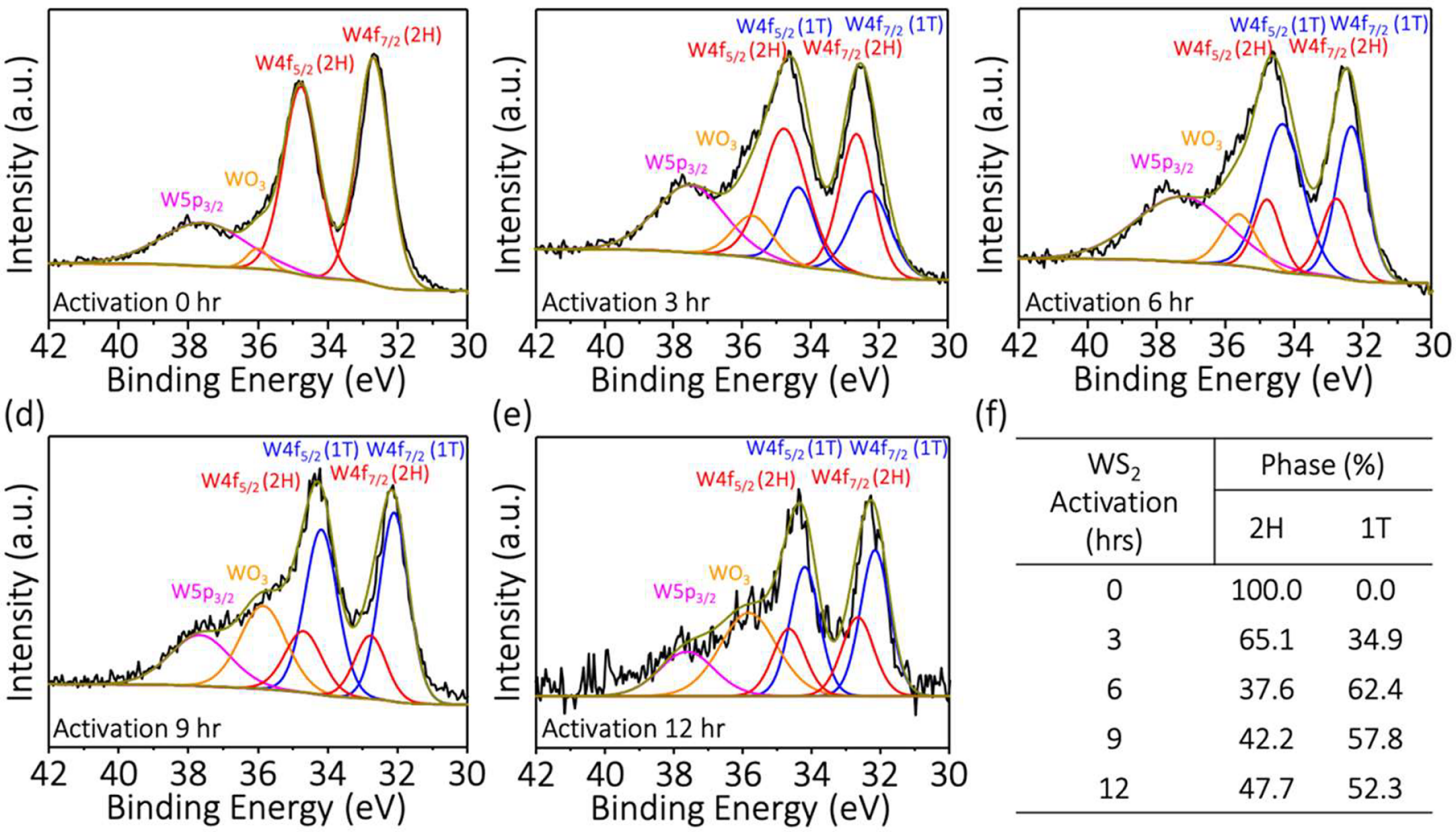
XPS characterization of 2H-WS_2_ and 2H-1T WS_2_. (a) 2H-WS_2_, (b) 2H-1T-WS_2_-3h, (c) 2H-1T-WS_2_-6h, (d) 2H-1T-WS_2_-9h, (e) 2H-1T-WS_2_-12h, and (f) summary of compositional phases analysis for samples.

To gain further insight into the electrocatalytic behaviors of 2H-1T-WS_2_ heterophase structures, electrochemical impedance spectroscopy (EIS) measurements were performed. Figure 6a shows the Nyquist plot of all 2H-1T-WS_2_ heterophase structures have smaller semicircles compared to as-prepared 2H-WS_2_ thin sheets, indicating low *R*_ct_ at the electrode–electrolyte interface. The *R*_ct_ of the 2H-1T-WS_2_-6h sample is only 15 ohm, which is much lower than that of 2H-WS_2_ thin sheets. Such a low *R*_ct_ value indicates that the converted 1T phase can efficiently improve the charge transfer process of the original 2H-WS_2_ in-plane structure. Besides, the formation of WO_3_ metal oxide may have impeded the charge transfer process to deteriorate the HER performance, as shown in Figure 6a. ECSA can be further utilized to elaborate the electroactivity of the same type of electrocatalyst. Figure S4 shows the CVs at different scan rates of the exfoliated 2H-WS_2_, 2H-1T-WS_2_-3h, 2H-1T-WS_2_-6h, 2H-1T-WS_2_-9h, and 2H-1T-WS_2_-12h samples. Remarkably, the ECSA of the 2H-1T-WS_2_-6h sample is 100 cm^2^, which is 20 times higher than the 2H-WS_2_ thin sheets (5 cm^2^) (Figure 6b). This suggests that in-plane 2H-1T-WS_2_ heterophase structures have many active sites due to 1T phase domain formations. Figure 6c shows the 2H-1T-WS_2_-6h sample exhibiting superior long-term stability from the 1st to 2000th LSV cycle, and only a limited difference can be observed.

**Figure 6:**
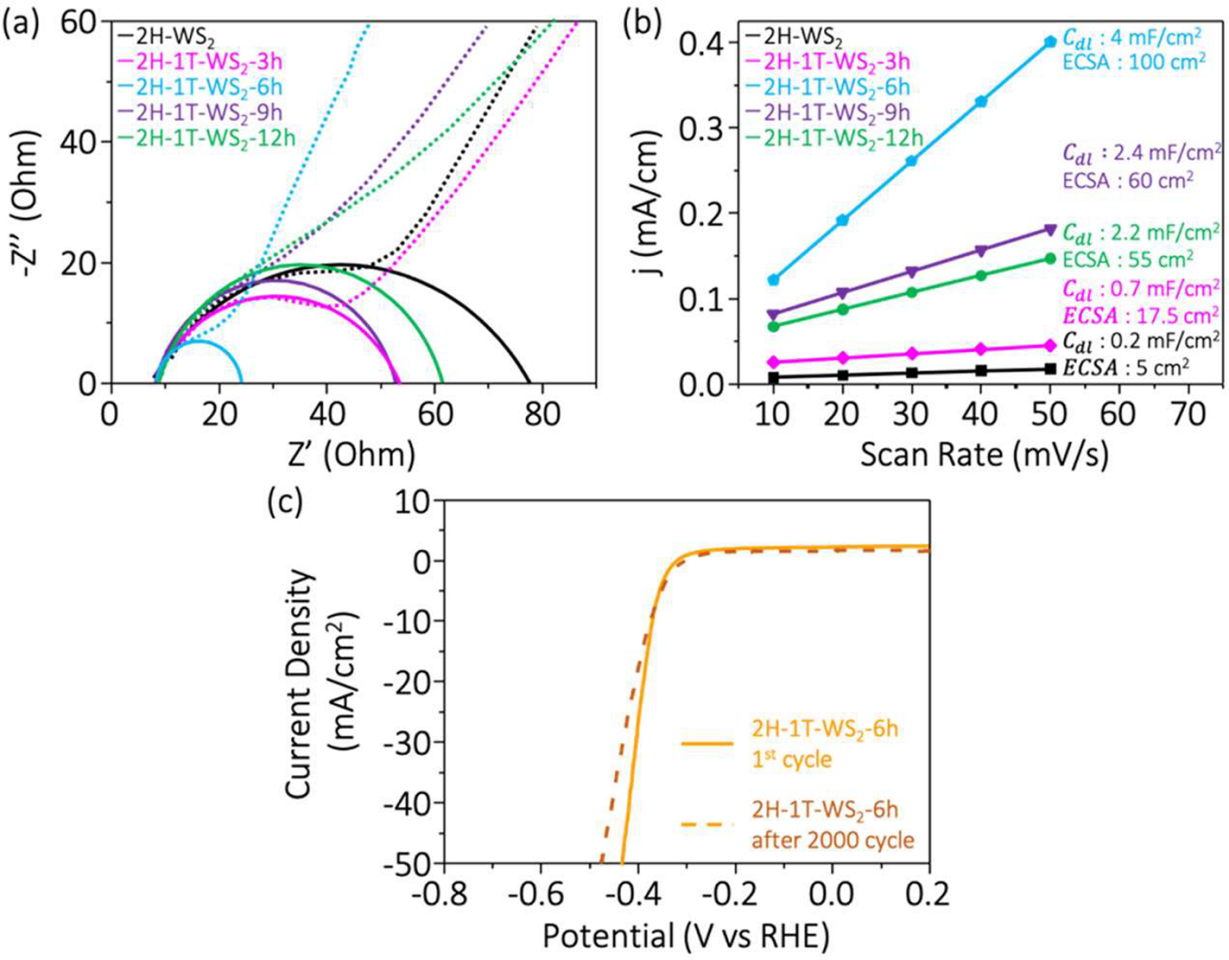
(a) Nyquist plots for 2H-1T-WS_2_ heterophase structures compared to the as-prepared 2H-WS_2_ thin sheets. (b) ECSA of catalyst samples. (c) Stability testing of the 2H-1T-WS_2_-6h sample.

According to the above structural analysis, the HER activity of the electrochemically activated 2H-1T-WS_2_ heterophase structures can be summarized in the following ways. First, the significantly modified electrical conductivity of the 2H-1T-WS_2_ heterophase structures enhance the electron transfer capability for HER. Second, there are lots of exposed active sites in the 2H-1T-WS_2_ heterophase structures, which is completely different from the catalytic inactive basal plane of 2H-WS_2_. Hence, the 2H-1T-WS_2_ heterophase structures exhibit significantly improved electrocatalytic and stability for HER, which agrees with the literature of mix phase of the TMDs.^7,29−31^

In conclusion, our study has demonstrated potentially induced structural modification as an effective method to fine-tune the catalytic properties of WS_2_ electrocatalysts. Through electrochemical activation, we observed the emergence of heterophase structures on the in-plane surface of WS_2_ thin sheets. These heterophases were achieved through a continuous transition from the 100% 2H phase to a balanced 2H:1T ratio via potential stimulation. Our findings offer valuable insights into phase changes in exfoliated thin sheets, paving the way for the preparation of heterophases in transition-metal dichalcogenides. This approach holds great promise for exploring phase-dependent properties, electrocatalysis, and applications in various electrochemical devices. We believe that our research contributes significantly to the advancement of electrocatalysis, making it a valuable addition to the field.

## Supporting Information Available

SEM images corresponding to the EDS spectrum and comparative table of weight percentages and atomic percentages; linear sweep voltammetry, cyclic voltammetry, and impedance curves (PDF)
