# Supplementary material for: Enhancing Hydrogen Evolution Catalysis through Potential-Induced Structural Phase Transition in Transition-Metal Dichalcogenide Thin Sheets
Source: J Phys Chem Lett. 2024 Feb 22;15(8):2287–92. doi: 10.1021/acs.jpclett.3c03305 (PMC10910572; doi:10.1021/acs.jpclett.3c03305)
Supplement: Supplementary file 1 — jz3c03305_si_001.pdf [file jz3c03305_si_001.pdf]

Supporting Information

Enhancing Hydrogen Evolution Catalysis through Potential-Induced Structural Phase Transition in Transition-Metal Dichalcogenides Thin Sheets

I-Wen Peter Chen[a], Yi-Lun Tseng[c], Jeremiah Hao Ran Huang[a], Kuan-Lun Chen[a],*

Tsai Yun Liu[b], and Jui-Chin Lee[b]

[a]Department of Chemistry, National Cheng Kung University, Tainan 701, Taiwan

[b]Core Facility Center, National Cheng Kung University, Tainan 701, Taiwan

[c]Department of Applied Sciences, National Taitung University, Taitung, 950, Taiwan

Methods

Chemicals.

WS_2 powder (99% metals basis; ~325 mesh powder) was purchased from Alfa Aesar. Sulfuric acid (H_2SO_4, 99.5%, HPLC grade) and acetone (HPLC grade) were purchased from ECHO chemical Co., Ltd. All chemicals were used as received.

Preparation of chlorophyll extracts.

Sapium sebiferum leaves (20 g) were ground in a mortar and pestle, then, 500 ml acetone poured into the mortar. After standing for one day, the leaves extracts were filtered through a polyvinylidene fluoride membrane (0.22 μm pore size) to remove impurities. After centrifuging for 30 min, the supernatant of the leaves extract solution was collected which is called chlorophyll extracts. The chlorophyll extracts concentration was ~ 5 mg/L.

Synthesis of 2H-WS_2 thin sheets.

According to previous established TMDs preparation method by our group, WS_2 powder (1 g), 1.6 mL of extracted chlorophyll solution (~ 5 mg/L), and 250 mL acetone were added to a 500 mL beaker at a controlled temperature, and sonicated with 100W sonication (Q700, Qsonica) in pulse mode for 5 h. To measure the concentration of the 2H-WS_2 suspension, 100 mL of solution was filtered through a 0.2 μm polyvinylidene fluoride membrane and the weight of the 2H-WS_2 measured. The suspension concentration of the exfoliated 2H-WS_2 thin sheets was ~1 mg/mL.

Electrochemical measurements.

HER experiments were measured using a CHI7279E electrochemistry workstation with a three-electrode system using 2 M H_2SO_4 as an electrolyte. In the three-electrode system, the chlorophyll-assisted exfoliated 2H-WS_2 thin sheets, Ag/AgCl and a graphite act as the working, reference, and counter electrodes, respectively. Take 10 μL of the 2H-WS_2 supernatant and drop it on the glassy carbon electrode

(GCE) and dry it. Then, the exfoliated 2H-WS_2 was applied a constant potential of -720 mV in 2 M H_2SO_4, and use chronoamperometry (i-t, Chronoamperometry) to activate the 2H-WS_2 for 3 h, 6 h, 9 h, and 12 h, and are denoted as 2H-1T-WS_2-3h, 2H-1T-WS_2-6h, 2H-1T-WS_2-9h, and 2H-1T-WS_2-12h, respectively. Linear sweep voltammetry (LSV) was measured at a rate of 1 mV/s. Cyclic voltammetry was measured at various rate to calculate the capacitance of the samples. Electrochemical impedance spectroscopy (EIS) was carried out in the frequency range from 10^{-2} Hz to 10^5 Hz at an amplitude of 5 mV and an applied potential of open circuit potential.

Characterization.

The images and energy-dispersive x-ray spectroscopy (EDS) of the WS_2 thin sheets were obtained using high-resolution transmission electron microscope (JEOL JEM-1400; JEOL-2100 CS STEM). The exfoliated 2H-WS_2 thin sheets were characterized by Raman spectroscopy with a 532 nm laser source, a laser power of lower than 100 mW and the signal of silicon at 520.7 cm^{-1} as a reference. The UV-Vis spectra were measured on a UV-Vis spectrophotometer (Unicam UV-300, Thermo Spectronic). Atomic force microscope (AFM; Innova/Bruker, Santa Barbara, CA) was utilized to measure the thickness of the exfoliated WS_2 thin sheets. The morphology of the composite paper was imaged by a field emission scanning electron microscope (FESEM; JEOL JSM-7600F). X-ray photoelectron spectroscopy (XPS) was performed using a PHI 5000 VersaProbe.

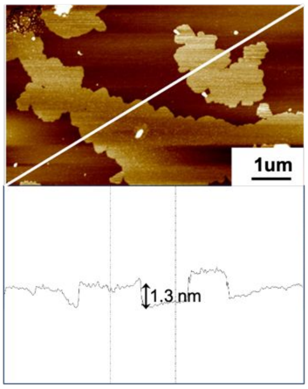

Figure S1. AFM image of the chlorophyll-assisted exfoliated WS_2 thin sheets.

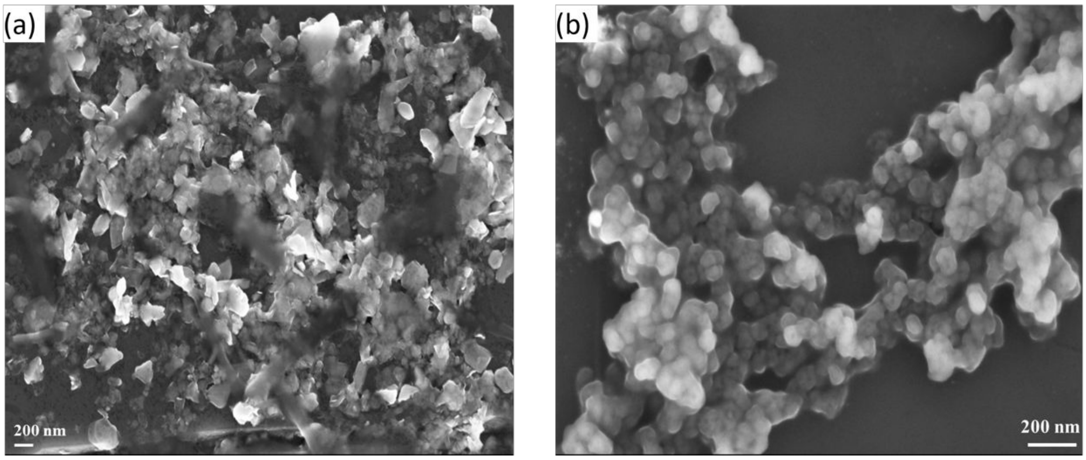

Figure S2. SEM image of a) exfoliated 2H-WS_2 thin sheets, b) 2H-1T-WS_2-6h sample.

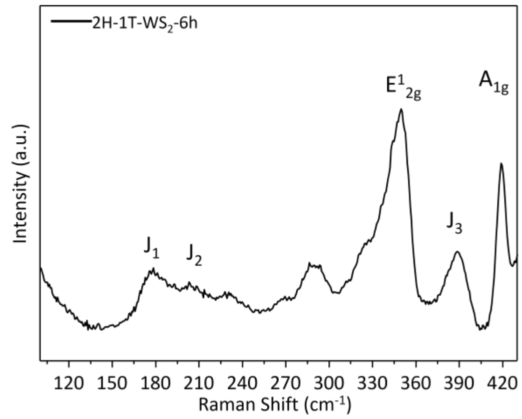

Figure S3. Magnification of the Raman spectrum of 2H-1T-WS_2-6h sample.

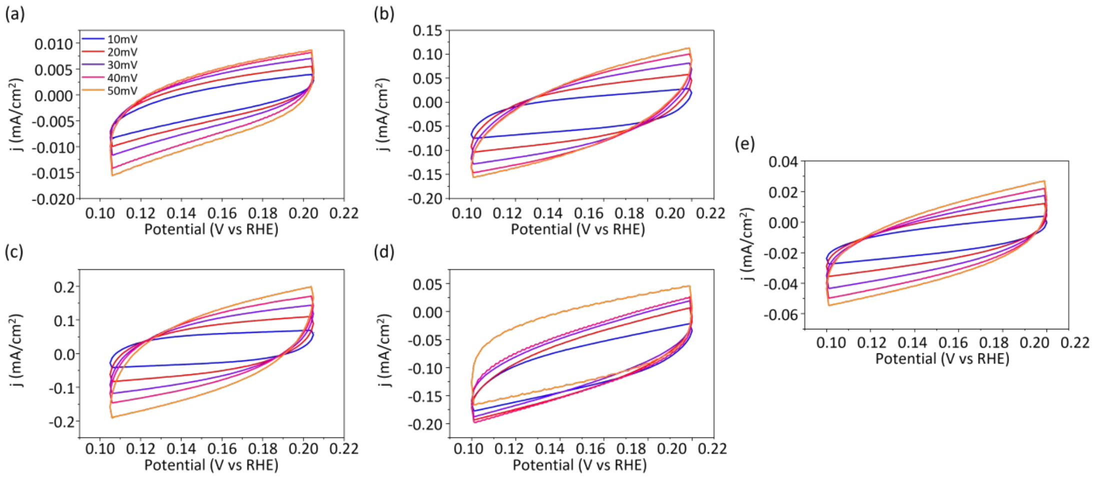

Figure S4. Electrochemical active surface area (ECSA) measurement at different scan rates of (a) exfoliated 2H-WS_2, (b) 2H-1T-WS_2-3h, (c) 2H-1T-WS_2-6h, (d) 2H-1T-WS_2-9h, (e) 2H-1T-WS_2-12h.

Table S1. TEM-EDS of the element composition of the 2H-1T-WS_2-6h sample.

Element	Atomic %
C(K)	81.8
O(K)	3.4
S(K)	7.0
Cu(K)	3.9
W(L)	3.7
